# Barriers to Use Artificial Intelligence Methodologies in Health Technology Assessment in Central and East European Countries

**DOI:** 10.3389/fpubh.2022.921226

**Published:** 2022-07-14

**Authors:** Konstantin Tachkov, Antal Zemplenyi, Maria Kamusheva, Maria Dimitrova, Pekka Siirtola, Johan Pontén, Bertalan Nemeth, Zoltan Kalo, Guenka Petrova

**Affiliations:** ^1^Faculty of Pharmacy, Medical University of Sofia, Sofia, Bulgaria; ^2^Syreon Research Institute, Budapest, Hungary; ^3^Center for Health Technology Assessment and Pharmacoeconomic Research, University of Pecs, Pecs, Hungary; ^4^Biomimetics and Intelligent Systems Group, University of Oulu, Oulu, Finland; ^5^Dental and Pharmaceutical Benefits Agency, Stockholm, Sweden; ^6^Centre for Health Technology Assessment, Semmelweis University, Budapest, Hungary

**Keywords:** artificial intelligence, barriers, Central and East European countries, health technology assessment, decision making

## Abstract

The aim of this paper is to identify the barriers that are specifically relevant to the use of Artificial Intelligence (AI)-based evidence in Central and Eastern European (CEE) Health Technology Assessment (HTA) systems. The study relied on two main parallel sources to identify barriers to use AI methodologies in HTA in CEE, including a scoping literature review and iterative focus group meetings with HTx team members. Most of the other selected articles discussed AI from a clinical perspective (*n* = 25), and the rest are from regulatory perspective (*n* = 13), and transfer of knowledge point of view (*n* = 3). Clinical areas studied are quite diverse—from pediatric, diabetes, diagnostic radiology, gynecology, oncology, surgery, psychiatry, cardiology, infection diseases, and oncology. Out of all 38 articles, 25 (66%) describe the AI method and the rest are more focused on the utilization barriers of different health care services and programs. The potential barriers could be classified as data related, methodological, technological, regulatory and policy related, and human factor related. Some of the barriers are quite similar, especially concerning the technologies. Studies focusing on the AI usage for HTA decision making are scarce. AI and augmented decision making tools are a novel science, and we are in the process of adapting it to existing needs. HTA as a process requires multiple steps, multiple evaluations which rely on heterogenous data. Therefore, the observed range of barriers come as a no surprise, and experts in the field need to give their opinion on the most important barriers in order to develop recommendations to overcome them and to disseminate the practical application of these tools.

## Introduction

Artificial Intelligence (AI) is the use of automated algorithms which perform processes, similar to the human brain's capacity to learn, synthesize, analyze, generalize, solve tasks etc. (1). Commonly, these algorithms are based on natural language processing, data mining, deep learning, machine learning (2). Natural language processing deals with accessibility of human language to machines and establishing the structure between words in a syntax, semantic, and pragmatic context. Data mining, on the other hand, deals with the conversion of unstructured to structured data (3) and machine learning, as a field, is concerned with the way machines acquire knowledge based on statistical or other type of informational inputs. Moreover, deep learning is a sub-field of machine learning which requires including huge amount of data in neural networks and thus increasing the computing power for processing complex datasets (4). There is little doubt that AI plays an important role for medical practice and science (5, 6), as more and more new medical devices and digital health solutions are supported by AI components. AI tools are widely used to assist individuals with health-related information; however, these are more limited to diagnostic and monitoring applications. The implication of AI has also been discussed in health economics and outcomes research, specifically in disease burden studies, drug utilization analyses, patient reported outcomes and comparative effectiveness research and economic evaluations (7, 8).

Health Technology Assessment (HTA) is a commonly used policy tool to assess the clinical and economic value of new technologies or existing technologies in new indications to support pricing and reimbursement decisions. From a welfare economics' perspective, the primary goal of healthcare policy is to maximize the health of the population from the limited resources available, within an ethical framework. Technology appraisal is a tool to substantiate this objective with the appropriate evidence base.

Research on the use of AI in healthcare is sparse, focused around specific clinical areas, or not systemized well-enough. Although AI methods for improving decision making have been investigated in detail since the late 80 s early 90 s (9, 10), the recent emergence of big data, and improvements in data collection, computing capacities and analytical methods as a concept offers more opportunities, which have yet to be fully investigated in the field of HTA.

In health care, with the increasing use of information systems and access to large amounts of data, the application of AI tools might facilitate the evidence base of policy decisions. Specifically, in the field of HTA, researchers can rely on health systems data such as administrative claims or electronic health records to generate evidence on health outcomes to support decisions of policy makers and inform patients about the utilization practice, effectiveness, or costs of technologies. Although the use of AI for generating evidence is promising, it has not been used widely in HTA.

This topic is particularly relevant for Central and Eastern European (CEE) countries, as HTA implementation is lagging behind Western European (WE) countries. The same also concerns the AI implementation in healthcare in all domains. Health care financing in CEE post-socialist countries is based on single payer systems, which creates an opportunity to follow patient pathways, explore comparative effectiveness data, and estimate resource utilization and cost of different diseases in a single national database (11). The national databases of health care payers have been digitalized in several CEE countries. AI can accelerate local evidence generation from these electronic databases and has the potential to reduce the gap between CEE and WE countries in HTA implementation. It could also make the decisions more transparent, and evidence driven, which is particularly important for the Central and Eastern European region.

Therefore, the aim of this paper is to identify the barriers that are specifically relevant to the use of AI-based evidence in HTA with special emphasis on Central and Eastern European HTA systems.

## Methodology

The study was conducted as part of the European Commission funded Horizon 2020 HTx project that is aiming to create a framework for the Next Generation Health Technology Assessment (HTA) to support patient-centered, societally oriented, real-time decision making on access to and reimbursement for health technologies throughout Europe.

The study relied on two main parallel sources to identify barriers to use AI methodologies in HTA in CEE, including a scoping literature review and iterative focus group meetings with HTx team members.

### Scoping Literature Review

A scoping review was conducted to explore barriers from the scientific and gray literature to identify a comprehensive list of issues of the use of AI in HTA and reimbursement decision making.

The MEDLINE database was accessed through PubMed and relevant articles, up to the 30th of September 2020 were identified through a combination of key words applied in different combinations: artificial intelligence OR machine learning) AND (implementation) AND (decision making) AND (health technology assessment; (artificial intelligence) AND (decision making); (healthcare) AND (decision making) AND (artificial intelligence); (artificial intelligence) AND (developing countries); (artificial intelligence) AND (developing countries) AND (health technology assessment); (artificial intelligence) AND (developing countries) AND (health technology assessment) AND (implementation). No other limits were introduced. We chose PubMed because it indexes references and abstracts on life sciences and biomedical topics.

**Figure 1:**
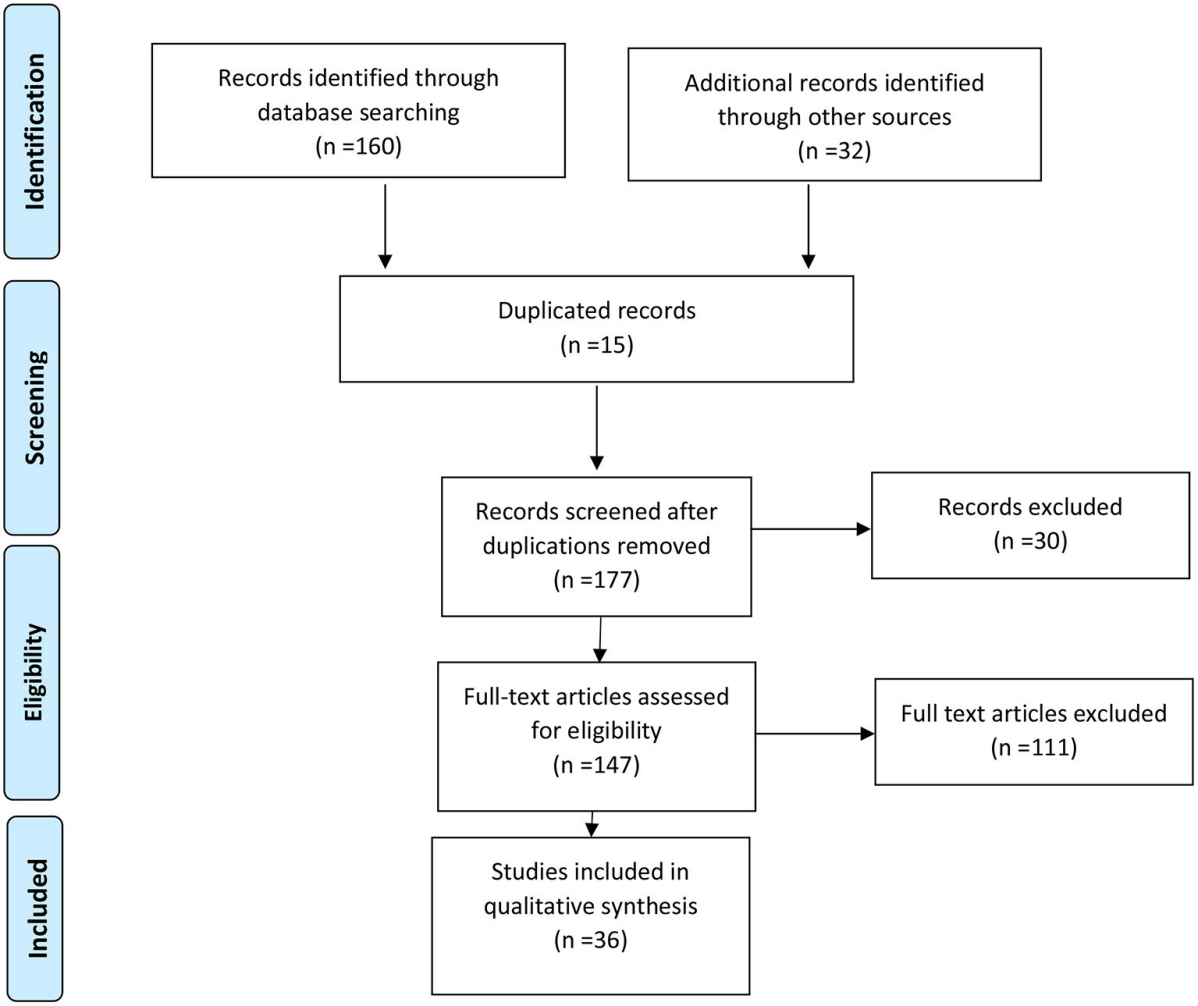
PRISMA diagram.

The articles found were reviewed by two independent reviewers for duplication and relevance to the study and topic criteria. We included those papers for full-text review which contained information on the use of AI for generating evidence in health care. Only English language articles were reviewed. A snowball method was also used to identify further relevant studies among the references of papers with full text review (12). Search flow is presented in the Prisma diagram (Figure 1) (13).

### Focus Group Meetings

In a series of iterative focus group meetings with HTx team members the list of barriers identified by the scoping review were reviewed, updated, and extended on a continuous basis. Permanent members of the focus group were recruited based on their multidisciplinary skills (e.g., medical, clinical epidemiology, data management and health economics) in relevant HTA sub-disciplines. Structured discussions were conducted about all issues where the experts had to judge which issues are relevant in the context of relying on AI driven evidence in HTA in CEE countries.

In general, the invited experts agreed that the barriers, outlined by the literature, are relevant for CEEC and that each country is trying to apply AI but mostly in unstructured, and rather unplanned order. Their opinion was that technological level and data obtaining methods differ significantly.

Identification of barriers from the literature and the focus groups were implemented in parallel by two different teams. After completion of their own research steps, the two teams merged the list of barriers, categorized them into five groups and resolved overlaps in the list of barriers.

### Expert Consultation

Subsequently as second focus group the initial list of barriers was reviewed by a group of 67 experts from Western, Central and Eastern Europe familiar with the HTA assessment methods and/or their application in the regulatory area in national context. The virtual workshop was organized in December 2020. Participants were identified in an iterative process relying on the professional networks of HTx partners. The main selection criteria were familiarity with the HTA and AI, with efforts for maintaining balanced geographical distribution of participants. The draft list of issues was presented to them, and they were asked to discuss the relevance of the identified barriers to the matter of interest, e.g., relying on AI for generating evidence for HTA and reimbursement decisions in Western or Central and East European countries. During these discussions the research team aimed to reduce overlaps in the list of barriers.

After the webinar a clarification of the terms used in formulating the barriers was made and final set was prepared for the publication.

## Results

### Results From the Scoping Review

Selected articles are presented in Supplementary Material 1 and titles of the excluded articles in Supplementary Material 2 with a reason for exclusion. The reasons for exclusion could be summarized as follows: general AI research (*n* = 43); topic not related to healthcare (*n* = 31); language limitation (*n* = 3); preclinical (*n* = 5); nationally oriented (*n* = 2); policy papers for different subjects (*n* = 5); therapeutic guidelines (3); position paper (*n* = 1).

Most of the other selected articles discussed AI from a clinical perspective (*n* = 25), and the rest are from regulatory perspective (*n* = 13), and transfer of knowledge point of view (*n* = 3). Clinical areas studied are quite diverse—from pediatric, diabetes, diagnostic radiology, gynecology, oncology, surgery, psychiatry, cardiology, infection diseases, and oncology.

Out of all 38 articles, 25 (66%) describe the AI method and the rest are more focused on the utilization barriers of different health care services and programs.

In the following section we offer a brief description of reviewed articles to outline the evolution of authors' thinking about possible future barriers. Regarding the AI method, 8 (32%) out of 25 articles focus on using the natural language processing method; 7 (28%) data mining, 10 (40%) used machine learning where 2 out of these 10 are using deep learning. The majority of articles focus on data mining, which offers the possibility of sifting through large datasets rapidly, indicating that collection and summary of data are some of the most important aspects of AI in generating evidence for HTA. Noteworthy, however, is the fact that language processing and machine learning tools are also frequently utilized, which underscores the complexity and multiple steps associated with manipulating patient information and patient data, especially with AI tools.

### Natural Language Processing

Natural language processing AI was used for supporting clinical decisions in pediatric intensive care (14, 15); to strengthen the hospital information system, i.e., to strengthen and standardize the data sharing aspect or data unification between different hospital information systems because data heterogeneity is an obstacle to the re-use of medical information. Chen (16, 17); to improve reporting of clinical trials results (18); health information archetypes building (19); supporting patients' care in cardiology for patients with language, visual or literacy impairment (20); educating people with infection diseases (21); to encode computer knowledge (22). These articles discuss good practices for standardizing data and improving databases that can enhance the use of AI-based methods. They also focus on the clinical aspect and application of health data, where AI can offer a systematic use of real-world evidence on effectiveness and safety to support treatment decisions. Effectiveness and safety are two important domains in the HTA decision making process, therefore the challenges presented in these use-cases are relevant for generalizing important barriers.

### Data Mining

Data mining for transformation of unstructured to structured data was used by Spaniga (23, 24) for specifying the patient's health history and predicting the results in diabetes care; Khan (25) for structuring information in the electronic patients' records; connecting databases in cardiology (26); creation of map-network in psychiatry (27). The articles by Spaniga and Fergherrazzi include data on quality of life for patients and algorithms to estimate future risk, applying the tool for future social burden of the disease, which again is a core domain of HTA. The other articles focus on structuring the data to help identify best clinical practices. When AI models are trained, the algorithms expect that input data is in certain format. Due to this, when unstructured data sources are used, a lot of manual work is normally required to process the data into a format that the AI trained on structured data understands. Information in unstructured notes is often recorded using very different terminology and abbreviations, making it difficult to automate the creation of metadata from unstructured data fields.

The article by Jabbour outlines the cost-saving aspect of unifying databases and addressing the needs of both patients and physicians in terms of the information they require. The article also highlights the fact that for certain healthcare systems, public investment might be required to improve standardized data collection. The articles discuss several methodological barriers to obtaining information from unstructured data sources.

### Machine Learning and Deep Learning

Machine learning was used in the area of diabetes to develop tools to routinely extract parameters and risk scores from row data and visualize them in descriptive manner (24, 26); in oncology for classifying purposes (28); in robotic surgery to review the machine learning techniques (29); in urology to standardize guidelines (30); in gynecology to predict clinical outcomes (31), in emergency departments (32). The deep learning AI was used in oncology for survival prediction (33) and in radiology diagnostics in processing medical images (34). These articles demonstrate use-cases for the application of AI to generate evidence from real-world data.

### Barriers Discussed in the Literature

Of the selected articles, 17 discussed the use of historically developed scientific, clinical and educational databases. These provide a valuable insight into the possible barriers to the introduction of the new technologies in the health care system, including the challenges associated with the processing and use of data as evidence, particularly in the context of assessing both the clinical and economic impact of these technologies. The work by MacCormac et al. (35) among the first, in this line, discussing the preparedness of people from developing countries to use the new health care technologies pointed out education as one of the most important barriers, as well as budgetary constraints and cultural differences.

The works of Basch (36) regarded the most impactful factors as being cultural, economic, social, institutional factors, as well as affordability, cost-effectiveness, and satisfying public demands. All other articles within this group are mostly concerned by the barriers in front the health technology assessment of new health technologies and their usage in the East European Countries jurisdiction (37–45). The barriers pointed out in the articles focused mainly on the methodological quality of documentation, differences in epidemiology and demographics, knowledge and differences in decision making criteria, differences in the organization of health care services delivery (46). The mentioned barriers could mean that, when trying to apply toward the health technology assessment, not only do the technical characteristics matter, but also differences in health care systems and peoples' perception toward these new technologies.

### Barriers Selected

The potential barriers discussed in the articles could be classified as data related, methodological, technological, regulatory and policy related, and human factor related (Table 1). Some of the barriers are quite similar, especially concerning the technologies and limitations of the databases.

**Table 1:** List of barriers to AI in HTA in CEE countries.

**Barriers**	
Data related barriers	Systemic bias in the data (e.g., due to upcoding)
	Issues with reliability, validity and accuracy of data (e.g., due to the lack of quality assessment of data entry or self-reporting)
	Raw fragmented or unstructured data (e.g., electronic medical records, imaging reports), which are difficult to aggregate and analyze
	Data acquisition and cleansing is not feasible
	Analysis of multicenter data is limited due to the lack of interoperability across systems (e.g., electronic medical records of different service providers)
	Lack of well-described patient level health databases
	Multinational data collection and analysis is limited due to differences in coding system across countries, and the lack of mapping methods to standardize the vocabulary
	Data that are relevant for research purposes are missing from databases built for the healthcare financing or provision (e.g., important clinical endpoints).
	The database is incomplete to fully track patient pathways, leading to inconsistent, unreliable findings
	Sample size of the available databases are low (e.g., databases of health care providers are not linked)
Methodological barriers	Potential bias of AI to favor some subgroups based on having more or better information
	Lack of transparency of protocols for data collection methods
	Text mining and natural language processing algorithms cannot be applied due to the lack of standardized medical terms in the local language
	Limited reproducibility due to the complexity of the methods
	Lack of methodological transparency of deep learning models (“black box” phenomenon)
	Complexity of the diseases and co-morbidities
Technological barriers	Lack of capacity to build and maintain IT infrastructure to support AI process
	High costs associated with securing and storing data for research purposes
	High cost of improving data validity (e. g. data abstracters to evaluate unstructured data)
Regulatory and policy related barriers	Regulatory compliance issues in the process of managing high volume of sensitive information
	Lack of awareness and openness on the part of decision-makers to rely on AI based real-world evidence
	Lack of political commitment (e.g., no health digitization strategy in the country to establish relevant databases)
	Acceptance and consent by patients and medical professionals
	Lack of access to patient-level databases due to data protection regulations
Human factor related barriers	Lack of knowledge in data governance: data ownership and data stewardship
	Lack of appropriate skills for applying AI methods (natural language processing, machine learning etc.) in outcomes research
	Lack of adequate education to generate AI driven scientific evidence
	Lack of decision-makers' expertise about the methods and use of AI driven scientific evidence

Despite the overlap between some of the barriers we found some important distinctions in detail. Overcoming data-related barriers appears to be the most difficult, as data is collected under different jurisdictions, protocols, coding, systems etc. This might negatively affect the possibility of data exchange and use it even for the same purposes. At this stage, the details like sample size, completeness of the datasets, maturity of the database could influence their reliability and validity not only at level of use but also at the level of decision making. In addition, the methodological barriers also contribute to the difficulties of data sharing with the potential biases, transparency issues of protocols, complexity of diseases, and applied algorithms.

Technological barriers are related to capacity of building IT structure like computer language and its capabilities, AI models and capacity of interpretation, as well as high cost of data curation and storage. All of them will probably play a significant role in the application of AI models to produce high-quality evidence in the health care system.

From technological, methodological and data transfer point of view, it seems to be important to standardize clinical practice and to document in a way that is understandable for the machines. Heterogeneity and complexity of the diseases, combined with a lack of interest toward novelties might also pose significant barriers to the acceptance of AI by clinicians. In this respect, it is not surprising that among the regulatory and policy related barriers, the willingness, awareness, commitment, and openness to adopt AI is major concerns not only for decision-makers, but also for patients and medical professionals.

Finally, the lack of expertise and knowledge on the technical, methodological and data governance issues demotivate people to rely on AI.

## Discussion

To the best of our knowledge this is one of the few articles diving deep inside the barriers related to the application of AI in HTA in CEE countries.

The scoping review revealed that numerous AI solutions exist, particularly in the era of “Big data” and are continuing their development (42, 47), however, choosing the best approach seems to be entirely dependent on the domain and the expertise of the people involved in this domain. Unlike other applied fields, HTA has several components—clinical, economical, ethical, legal, and socio-cultural where as recently as 2018 the need for standardized good practices was identified (48). The first hurdle lies in viewing AI technology tools as assistants and support systems for decision making, the second lies in choosing the precise tool, for the correct aspect of HTA. This paper is an attempt to generate a wider discussion within the whole HTA community and especially that from CEE countries on this particular topic, and to raise awareness for the need of collaboration between different fields like medical and computer science. The aim of this paper was to identify the main barriers to the use of AI in HTA. We have not focused on recommendations to address these barriers as this will be published in a separate paper.

As was revealed, decisions can be structured (based on deterministic data, requiring almost no human input), unstructured (relying exclusively on the decision maker) or somewhere in between as “semi-structured.” In our view, the HTA community should initiate conversation around this aspect and build on these discussions to explore best practices for relying on AI-based evidence in initial assessments and make recommendations on how big data can generate more accurate inputs for re-evaluating technologies. However, the second point is a subject of further research and additional input is required from experts in the field on how to prioritize barriers and what recommendations can be made to address and overcome them.

Artificial intelligence presents a challenge for health care researchers when they need to demonstrate its application in predicting diseases development, health outcomes and how this information can be shared and related to different clinical settings and even countries (49). It is also a challenge for policymakers who need to interpret such evidence and apply their results in evaluating the introduction of new technologies into national markets, a process compounded by the complexity and the required level of understanding (50, 51).

Some authors argue that AI will become more widespread in HTA and offer novel, revolutionary mechanisms by automating routine tasks, broadening access, targeting more precisely patient needs (52), however, Central and Eastern European countries still lag behind their western counterparts in terms of information systems and application of novel technologies. Despite sharp technological developments, there is a strong digital divide between urban and rural areas, as well as a slow political process when introducing new technologies (53). Policy decisions require thorough research, detailed description of problem areas and potential solutions, tailored to societal needs. Thus, identifying the barriers to the use of AI in HTA that policy makers may encounter offers a starting point for a discussion centered around finding probable regulatory solutions. Further studies are needed to rank the barriers, identified in our study, according to their importance and relevance to Central and Eastern European context and to discuss regulatory measures to overcome the most important barriers.

Taking into consideration the definition of “decision making,” we posit that the judgements on safety and effectiveness can be considered as structured decisions, judgements on cost-effectiveness are semi-structured decisions due to the wide range of requirements of different HTA bodies (54), whereas judgements on social and ethical implications are fully unstructured decisions. Discussion on where AI fits within this domain is purely theoretical, but two seminal moments are worth mentioning. Firstly, AI systems, since their inception, have been considered purely as augmenting tools, helping human intelligence (55). This means that human input and expertise is paramount when designing a decision making support system in order to find the best approach, since there's a broad range of options. Secondly, HTA standardization is still in its infancy and the current impact of big data has yet to be discussed broadly in the literature.

Due to its rapid recent development, AI has the potential to revolutionize healthcare decision making. AI models are based on data-driven processes, and as a results of this AI can find highly complex and novel connections from the data that are not visible to the human eye. Specifically in HTA, AI has the potential to process large amounts of real-world data to generate real-world evidence, which has become increasingly important in evaluating the clinical effectiveness and cost-effectiveness of new technologies in real life.

The barriers reviewed here have pointed out that there is still a long way to go before HTA can benefit from the AI advances in all European countries. Nevertheless, we should acknowledge, that the topic of this study is a new area of scientific inquiry. Many of the barriers addressed, such as data-related issues, language barriers and difficulties in data sharing are common among all European countries, and not just in the field of healthcare. The European Union Agency for Cybersecurity has outlined the barriers of high and low importance for data sharing in general in their 2010 report—Incentives and barriers to data sharing (56). A recent study by Bentzen et al. has emphasized the importance of health data in furthering research (57).

We found that relatively limited number of articles discussed the AI predictions to health economic and HTA decision (43, 46–51). One possible reason could be that the HTA bodies need consistent and structured information for decision making and there are no standards how to systemize existing knowledge in the way that it will benefit the decision making process.

The work of (58) had a slightly different approach but led to the same conclusions. Authors decided to use the structure of the EUnetHTA Core models and to identify the key challenges posed by AI. Some of the key changes were also formulated as barriers in the current study despite the fact both studies were developed completely independent. The authors highlighted the need for HTA scholars and practitioners to explore methods that complement traditional data collection and information gathering that can better inform AI-based decisions. They suggest that AI-based systems should be seen as a tool for transforming the healthcare system rather than as a separate set of technological tools. They propose that “creating the political, regulatory, organizational, clinical, and technological conditions necessary for proper innovation is the first step” to guide the development of AI, generate knowledge and draw actionable lessons.

According to GDPR, automated processes that use personal data, like AI, must be able to explain the reasons behind the outcome. However, the decisions made by AI can be based on complex underlying mechanisms which can be difficult, or even impossible, to explain. Another problem is that AI models do not understand causality, due to this, the data used to train AI models and the models themselves need to be analyzed together with domain experts to avoid misleading results. The reproducibility of AI analyses may be limited by the complexity of the methods and the lack of methodological transparency of deep learning models. This limits the transferability of the method to other datasets in different jurisdictions.

AI is a tool and like any other tool, you can use in a right way or a wrong way. Currently, AI is not smart enough to make decisions itself, but should be used a tool for medical experts to improve decision making when optimal treatment for patient is chosen. Also in HTA, the best available evidence should be used to support a reimbursement decision. Artificial intelligence, applied in a transparent way, can be very useful, for example, in generating evidence on the relative effectiveness of a treatment or in determining which group of patients a therapy might be more effective for. It is a tool to support the reimbursement decision, not replace it.

A limitation of our study was that we only worked from public databases and did not have access to repositories of HTA dossiers where we could review cases where AI has been used to demonstrate safety, effectiveness or cost-effectiveness. The discussion with the experts was limited to identifying the barriers, rather than prioritizing them and proposing possible solutions, which will be a planned step in a future study.

## Conclusion

Studies focusing on the AI usage for HTA decision making are scarce. Barriers to the use of AI in HTA in CEE countries are diverse and consist of methodological, technological, regulatory and policy, human factor, and data related issues.

AI and augmented decision making tools are a novel science, and we are in the process of adapting it to existing needs. HTA as a process requires multiple steps, multiple evaluations which rely on heterogenous data. Therefore, the observed range of barriers come as a no surprise, and experts in the field need to give their opinion on the most important barriers in order to develop recommendations to overcome them and to disseminate the practical application of these tools.

## Data Availability Statement

The original contributions presented in the study are included in the article/Supplementary Material, further inquiries can be directed to the corresponding author/s.

## Author Contributions

KT, ZK, and GP: conceptualisation. KT, MD, MK, and BN: data collection. KT, BN, MD, MK, GP, AZ, ZK, PS, and JP: data validation. KT, AZ, MD, MK, GP, ZK, PS, and JP: data analysis, writing first draft, and final version writing and approval. All authors contributed to the article and approved the submitted version.

## Funding

The HTx project has received funding from the European Union's Horizon 2020 research and innovation programme under grant agreement no. 825162. This dissemination reflects only the author's view and the Commission is not responsible for any use that may be made of the information it contains.

## Conflict of Interest

The authors declare that the research was conducted in the absence of any commercial or financial relationships that could be construed as a potential conflict of interest.

## Publisher's Note

All claims expressed in this article are solely those of the authors and do not necessarily represent those of their affiliated organizations, or those of the publisher, the editors and the reviewers. Any product that may be evaluated in this article, or claim that may be made by its manufacturer, is not guaranteed or endorsed by the publisher.
