# Supplementary material for: Barriers to Use Artificial Intelligence Methodologies in Health Technology Assessment in Central and East European Countries
Source: Front Public Health. 2022 Jul 14;10:921226. doi: 10.3389/fpubh.2022.921226 (PMC9330148; doi:10.3389/fpubh.2022.921226)
Supplement: Supplementary file 1 [file Table_1.pdf]

Supplementary material 1. Articles included in the barrier selection

Authors	Area of application	Therapeutic and or another sphere	AI method	Transferability barriers discussed
Hein J W. (1986)	Discussion paper		No AI	Demand for technological responsibility and democratic responsiveness as the fundamental problem of science and policy-making in democratic societies.<br>A statement in a letter to the Editor to the British Medical Journal in 1984 succinctly illustrates much of what has gone wrong in medical technology transfer to underdeveloped nations. The writer pointed out that they stopped sending tuberculosis patients from their small rural hospital in Zaire to the large hospital in the capital when it was discovered that, having spent all their money on expensive top-quality radiographs and drugs, the referred patients promptly died of malnutrition. I have deliberately avoided the mention of dentistry up to this point because I believe a brief historical summary of the evolution of the concept of technology transfer and the difficulties encountered through its application in other areas would provide a useful perspective for judging what forms of technology transfer would be most useful for our profession to implement.
Kahen G (1997)	Knowledge transfer	Cardiology	NPL - Analytic Hierarchy Process	Exchange of experiential information - Exchange of experience in clinical or public health issues and access to medical advances Information on locally applicable and cost-effective research and discoveries<br>Raising awareness - engage professionals and the public and sensitize them to prevention<br>Advocacy for progressive policies - Forming alliances to pressure for prevention-oriented national policies<br>Access to epidemiologic data - Electronic surveillance and databases on cardiovascular diseases and their risk factors<br>Training and research - Distance learning and access to expertise in conducting essential research<br>Local and international networking (South – South as well as North – South networking)

Perry S (1992)	Regulatory	Establishment of HTA structure	No AI	Lack of technical personnel in institutions<br>Difference in the structure of disease prevalence and mortality reasons<br>Lack of preventive programs<br>In developing countries there are seldom adequate funds for the creation of a separate entity for health technology<br>The cost of operating and maintaining equipment is often much higher in developing countries
Mack EH (2009)	Clinical	Pediatric intensive care	NPL – CDSS	Liability, human factors engineering, alert fatigue, and audit trails.
Wulff A. et all. (2018)	Clinical; technical practicability of real clinical data	Pediatric intensive care	NPL - Clinical decision-support systems (CDSS), combining openEHR Archetypes, terminology bindings and the Archetype Query Language (AQL)	Computer language;<br>Quality of data;<br>Suitable reference AI model;<br>Accurate clinical stages description;<br>Guideline definition language – weaknesses in handling and performance;
Chen R., (2009)	Regional to Open electronic health records	Informational	NPL - OpenEHR EHR Reference Model (RM) and Archetype Model (AM)	Exchange of Electronic Health Record (EHR) data between systems from different suppliers;<br>Semantic interoperability between different EHR systems across technical platform;<br>Development of standard based clinical models.
Kopanitsa G. (2017)	Hospital information system	Informational (Electronic health record data)	NPL - Clinical decision support systems (CDSS) interacting with electronic health records	Heterogeneity of health records systems;<br>Defining queries to a valid data set with known structure and constraints.
Marcos M., (2013)	Clinical trials	Regulatory	NPL - CDSSs and EHRs based on archetypes	Standardization of the virtual health records;<br>Mapping source EHR data to the CDSS;<br>Heterogeneous formats, models, abstractions levels, and semantics.

Wollersheim D (2009)	Literature review	Health information	NPL - Archetypes for Health Information Managers.	Archetypes are an appropriate solution for future-proof and interoperable medical data storage (only summary)
John D Piette (2015)	Clinical	Cardiology	NPL - mHealth apps	Because no reading or writing is required, IVR (interactive voice response) interactions are accessible to low-literacy populations as well as those with vision problems<br>Provide ongoing patient care at a distance<br>Language barriers<br>New systems should consider behavioral theory.
Hendra van Zyla (2010)	Educational, knowledge transfer	HIV	NPL - Information and Communication Technologies (ICT) platforms	Educating audiences on a disease; updating audiences on current issues and developments; facilitating decision-making; improving peer interaction, and; stimulating innovative knowledge transfer, with the latter focusing on innovative online as well as community outreach projects.<br>Identify the target audiences; • Ascertain whether the knowledge intended for the audience is required by them; • Classify content according to the health literacy of the audience; • Show the relevance of new knowledge; • Avoid an information overload; • Strive for utilisation of knowledge by the audience; • Address the issues relevant to the audience.<br>Selection of appropriate push and pull technologies to transfer and share knowledge; Packaging content with cultural understanding; Classifying content for different audiences; Packaging content according to Web Accessibility standards; Applying a convergence of ICTs to address the information needs of consumers; Implementing methodologies to measure health consumers' knowledge levels and packaging content accordingly; Implementing content to prevent an information overload; Implementing intuitive and easy navigation; Implementing quality assurance methodologies such as the HON principles3 , workflow processes, and an editorial process; • Implementing policies, terms of reference for use, explanations and assistance.
Jenders RA (2003)	Knowledge transfer	Medical information	NLP - Arden syntax to encode computable knowledge	Delay often between confirmation of a clinically relevant research finding in the medical literature and the incorporation of that finding into widespread clinical practice;<br>Lack of awareness, lack of familiarity and inertia of previous practice;

Späniga S. et al. (2019)	Clinical and educational; AI able to interact with a patient (virtual doctor),	Type 2 diabetes	DM- Automated, interactive anamnesis with a non-invasive T2DM prediction. The virtual doctor is a cabin with several devices to obtain patient metrics. The embedded AI utilizes these metrics, such as the patient's BMI, to identify possible diseases or impairments to health. Finally, the AI recommends further diagnostic steps to the medical personnel, such as the HbA1c blood test	Acceptance and consent by patients and also by medical professionals;<br>Educational status and age of people;<br>Matters of data storage, data transfer, and data integrity compliant with respective laws of data protection in countries willing to implement such a system;
Fagherazzi G (2019)	Clinical	Diabetes	ML - All diabetes technology facilities	The development of tools to routinely extract parameters and risk scores from raw data and visualize them in a descriptive manner;<br>Evidence-based guidelines provided by learned diabetes societies;<br>Patient empowerment evoked by digitization of care will certainly place patients at the center of disease management and research;<br>Need to train patients and caregivers;<br>Balance between benefits and risk of data privacy exploitation, and the impact of digital medicine on patients, caregivers, care organizations;<br>Patients informed consent in research should be transformed;<br>No standard protocols for data exchanges and interoperability;

				Limiting the risk of data or technological hacking and maintaining the trust of users for new technologies;<br>Availability of new technology does not equate to acceptability by all;<br>The use of digital tools in diabetes should serve to reduce social inequities, while taking great care not to increase the already existing digital barriers to healthcare and treatment innovations by socially disadvantaged populations;<br>To limit biases, discrimination against and underrepresentation of specific groups of populations AI algorithms is essential to expand the diversity of participants in epidemiological and clinical studies.
Khan Muhammad AH (2019)	Regulatory	Electronic patients records and consultation system	DM - District Health Information Software 2 (DHIS2)	Strong support at the highest political level;<br>Data standardization and interoperability in eHealth software<br>Strategies for building capacity to develop health-related big data applications.<br>Shortages of human resources.
Jabbour S. (2003)	Clinical	Cardiology	DM - Informational databases as ProCOR, Global Cardiovascular Infobase, Heartfile, and the Virtual Congress of Cardiology	Cost, feasibility, and relevance of information need to be considered before wide adoption is advocated;<br>Widening global information gap, inequitable access, and irrelevant information. For now, information technology must be viewed as part of a broader strategy, which includes conventional communication media, to address the unmet information needs
Chung Y. (2018)	Clinical and regulatory	Psychiatry	DM - Self-organising map network (SOMNet)	Small number of end-users with sufficient knowledge;<br>Necessity of a longitudinal and corroboration studies to validate the usability of models.<br>Necessity of adding a model assessment stage in its post-processing of data for further expert-guided data analysis
Frank P Y Lin (2016)	Clinical	Oncology (breast cancer)	ML - Machine learning classifiers with and without bootstrap aggregation	Require a multidisciplinary approach;<br>Machine learning models were high discriminative of the outcome variables (like patient reported outcomes)
Kassahun Y (2016)	Clinical	Robotic surgery	ML - Review of ML techniques	Classification and standardization of medical practice;<br>Limited use in current surgical practice;

				Need of large quantities of high-quality medical and surgical data;<br>To come up with metrics that adequately capture the characteristics of best practice;<br>Adaptation to unknown or yet unobserved situations;<br>Need for a structured approach to efficiently transfer surgical skill toward automated execution.
Papageorgiou E (2012)	Clinical and regulatory (guideline)	Urology	ML - Fuzzy Cognitive Maps (FCMs) and semantic web approach.	Depends on the number of guidelines selection;<br>Depends on the complexity of the disease and co-morbidities.
Uyar A (2015)	Predicting clinical outcomes	Gynecology	ML - naïve Bayes model	Data base availability and reliability<br>Clinical techniques used
Yoshihiko R. (2019)	Clinical	Emergency care	ML - Machine learning models - Lasso regression, random forest, gradient boosted decision tree, and deep neural network	Quality of data is important in data-driven machine learning-based prediction;<br>Missing information;
López-García G. (2020)	Survival prediction	Oncology, genetics	DL - Deep learcing - Convolutional networks	Unstructured nature of data from disease sample;<br>Model interpretability,<br>Most of DL models are still considered as "black-boxes";.
Ahmed H. (2018)	Clinical	Radiology diagnostic	DL - Deep learning applications in medical imaging	Need for standardized data collection methods, evaluation criteria, prospective validation, and reporting protocols;<br>Non transparent type of deep learning methodology.
MacCormack CP (1989).	Psychological	Different disease areas	Different technologies	1) numbers of women health workers trained by grade of job; 2) the time-saving potential of improved technologies through women's time budgets; 3) technology transfers to document women's control of the technology 5 years after the end of the project; 4) the range of medical technologies being offered, compared with user preference; 5) the proportion of women trained to maintain health-related technologies; 6) the proportion of self-help appropriate technologies compared with specialist technologies, and 7) when

				medical technologies/practices are not widely used, are programs redesigned to be more culturally and gender appropriate and economically feasible?
Basch PF. (1993)	Regulatory	Technology transfer	No AI	Technologic novelty is far less important than relevance, which encompasses, among other things: direct application to reducing risk of infection and disease; affordability and cost-effectiveness; saving foreign exchange; satisfying public demand with political benefit to the government; and promotion of social equity.
Pichon-Riviere A. (2012)	Regulatory	HTAs DM transfer	No AI	poor methodological quality,<br>different epidemiological context.
Mandrik O. (2015)	Regualtory	HTAs transferability	No AI	Uncertainty, impact of influential parameters, and data transferability.<br>Limitations of foreign data use<br>Information (data and knowledge) constraints
Saarni SI (2008)	Regulatory	HTA ethical issues	EuNetHTA package	Can technology challenge the religious, cultural or moral convictions or beliefs of some groups or change current social arrangements?<br>Does the implementation or use of the technology challenge patient autonomy?
Sideman S, (1997)	Regulatory	HTA	No AI	Less developed countries (LDCs) are limited in medical resources. Management talents are scares<br>Significant economic, political, and ethical ramifications.
Németh Bertalan (2018)	Clinical	Prevention program	No AI – Utility model	Transferring evidence requires context-specific data to go into the model;<br>Limited resources and time in case of lack of data;<br>Specific set of indicators was developed to describe the transferability barriers strongly connected to prevention program.
Kulchaitanaroaj P. (2018)	Clinical	Prevention and CEA	No AI	Possible disagreement between the stakeholder opinion and CEA results;<br>Importance of previous beliefs and perceptions of stakeholders;<br>Strong engagement of stakeholders is needed;<br>Limited resources for preventive educational measures and replacement therapy.
Zoltán Kaló (2016)	Regulatory	HTA	No AI	Country-specific aspects should be considered, such as country size, gross domestic product per capita, major social values, public health priorities, and fragmentation of healthcare financing.

				Capacity building, financing HTA research, process and organizational structure for HTA, standardization of HTA methodology, use of local data, scope of mandatory HTA, decision criteria, and international collaboration in HTA.
Zoltán Kaló (2020)	Clinical	Integrated care model	No AI	Identify potential barriers to implementing integrated care models; Prioritize the identified barriers; Generate consensus among multiple local stakeholders on local feasibility and on potential solutions to key barriers; Publish findings to extend the evidence base; Select evidence-based models; Proven benefits should be relevant in the local context; Estimate the magnitude of potential benefits in the local context; Policy-relevant methodology; Consistent and transparent decision rule; Continuous performance monitoring of implemented models; Locally relevant financing methodology; Long term financing.
Michael Drummond (2015)	Regulatory	HTA	No AI	Differences in current standard of care, practice patterns, or gross domestic product between the developed countries;<br>Differences in performed studies<br>Differences in jurisdictions.
Nicod E. (2016)	Regulatory	HTA coverage decisions	Sequential exploratory mixed methods research approach was used to develop and pilot the methodological framework in the form of an instrument development design.	Transferability to other countries and therapy areas is limited to those cases where similar decision-making criteria are accounted for HTA entities that are arm’s length, responsible for issuing coverage recommendations, and have a transparent process where sufficient detail about the appraisal process and reasons for the final decision are recorded in their decision reports.
