# Supplementary material for: Barriers to Use Artificial Intelligence Methodologies in Health Technology Assessment in Central and East European Countries
Source: Front Public Health. 2022 Jul 14;10:921226. doi: 10.3389/fpubh.2022.921226 (PMC9330148; doi:10.3389/fpubh.2022.921226)
Supplement: Supplementary file 2 [file Table_2.DOCX]

**Supplementary material 2. List of Excluded articles and reasons for removal**

Saria S, Butte A, Sheikh A (2018) Better medicine through machine learning: What’s real, and what’s artificial? PLoS Med 15(12): e1002721. <https://doi.org/10.1371/journal.pmed.1002721> (position paper)

Victor J Barranca, Han Huang, Genji Kawakita. Network structure and input integration in competing firing rate models for decision-making. J Comput Neurosci. 2019 Apr;46(2):145-168. doi: 10.1007/s10827-018-0708-6. (do not discuss the implementation in health care)

Liheng Gong, Xiao Zhang, Ling Li. An Artificial Intelligence Fusion Model for Cardiac Emergency Decision Making: Application and Robustness Analysis. JMIR Med Inform. 2020 Jul 27;8(7):e19428. doi: 10.2196/19428. (Theoretical article)

Feng Li, Hua Chen, Zheng Liu, Xuedian Zhang, Zhizheng Wu. Fully automated detection of retinal disorders by image-based deep learning. Graefes Arch Clin Exp Ophthalmol. 2019 Mar;257(3):495-505. doi: 10.1007/s00417-018-04224-8 (do not discuss the transferability)

Farheen Ramzan, Muhammad Usman Ghani Khan, Asim Rehmat, Sajid Iqbal, Tanzila Saba, Amjad Rehman, Zahid Mehmood. A Deep Learning Approach for Automated Diagnosis and Multi-Class Classification of Alzheimer's Disease Stages Using Resting-State fMRI and Residual Neural Networks. J Med Syst. 2019 Dec 18;44(2):37. doi: 10.1007/s10916-019-1475-2 (do not discuss the transferability)

Liliya Serazetdinova, James Garratt, Alan Baylis, Sokratis Stergiadis, Martin Collison, Simon Davis. How should we turn data into decisions in AgriFood? J Sci Food Agric. 2019 May;99(7):3213-3219. doi: 10.1002/jsfa.9545. (not related to health care)

Rose L Molina, Matthew Gombolay, Jennifer Jonas, Anna M Modest, Julie Shah, Toni H Golen, Neel T Shah. Association Between Labor and Delivery Unit Census and Delays in Patient Management: Findings From a Computer Simulation Module. Obstet Gynecol. 2018;131(3):545-552. doi: 10.1097/AOG.0000000000002482. (do not discuss the transferability)

Philipp Berens 1 2, Sebastian M Waldstein 3 4, Murat Seckin Ayhan 5, Louis Kümmerle 5, Hansjürgen Agostini 6, Andreas Stahl 7, Focke Ziemssen. Potential of methods of artificial intelligence for quality assurance. Ophthalmologe. 2020 Apr;117(4):320-325. doi: 10.1007/s00347-020-01063-z. [Article in German]

Xiaoyu Tan, Yonggu Lee, Chin-Boon Chng, Kah-Bin Lim, Chee-Kong Chui. Robot-assisted flexible needle insertion using universal distributional deep reinforcement learning. Int J Comput Assist Radiol Surg. 2020 Feb;15(2):341-349. doi: 10.1007/s11548-019-02098-7. (no transferability issue)

Kwokwing Chau. A review on the integration of artificial intelligence into coastal modeling. J Environ Manage . 2006 Jul;80(1):47-57. doi: 10.1016/j.jenvman.2005.08.012. (do not refer to health care)

Miguel Gonzalez, Richard Watson, Seth Bullock.Minimally Sufficient Conditions for the Evolution of Social Learning and the Emergence of Non-Genetic Evolutionary Systems. Artif Life. Fall 2017;23(4):493-517. doi: 10.1162/ARTL_a_00244. (preclinical)

Afsardeir A, Keramati M. Behavioural signatures of backward planning in animals. Eur J Neurosci. 2018 Mar;47(5):479-487. doi: 10.1111/ejn.13851. (preclinical)

Chung YH, Lee T, Yoo SY, Min J, Choi JW. Electrochemical Bioelectronic Device Consisting of Metalloprotein for Analog Decision Making. Sci Rep. 2015 Sep 24;5:14501. doi: 10.1038/srep14501. (not in health care domain)

Zhuo Wang, Andrew T Sornborger, Louis Tao. Graded, Dynamically Routable Information Processing with Synfire-Gated Synfire Chains. PLoS Comput Biol. 2016 Jun 16;12(6):e1004979. doi: 10.1371/journal.pcbi.1004979. eCollection 2016 Jun. (no health care)

Amr Farahat, Christoph Reichert, Catherine M Sweeney-Reed, Hermann Hinrichs.Convolutional neural networks for decoding of covert attention focus and saliency maps for EEG feature visualization. J Neural Eng . 2019 Oct 23;16(6):066010. doi: 10.1088/1741-2552/ab3bb4. (no health care)

Amin Mousavi, Babak Nadjar Araabi, Majid Nili Ahmadabadi. Context transfer in reinforcement learning using action-value functions. Comput Intell Neurosci. 2014;2014:428567. doi: 10.1155/2014/428567 (no health care)

Rodríguez-Muñiz LJ, Bernardo AB, Esteban M, Díaz I. Dropout and transfer paths: What are the risky profiles when analyzing university persistence with machine learning techniques? PLoS One. 2019 Jun 21;14(6):e0218796. doi: 10.1371/journal.pone.0218796. eCollection 2019. (no health care)

Sakai Y, S Takemoto, K Hori, M Nishimura, H Ikematsu, T Yano, H Yokota. Automatic detection of early gastric cancer in endoscopic images using a transferring convolutional neural network. Conf Proc IEEE Eng Med Biol Soc.;2018:4138-4141. doi: 10.1109/EMBC.2018.8513274. (no transferability)

Chase E Thiel, Shane Connelly, Lauren Harkrider, Lynn D Devenport, Zhanna Bagdasarov, James F Johnson, Michael D Mumford. Case-based knowledge and ethics education: improving learning and transfer through emotionally rich cases. Sci Eng Ethics. 2013 Mar;19(1):265-86. doi: 10.1007/s11948-011-9318-7 (no health care)

Ralf Holzer 1, Ed Ladusans, Denise Kitchiner, Ian Peart, Gordon Gladman, Gail Miles Prioritization of congenital cardiac surgical patients using fuzzy reasoning--a solution to the problem of the waiting list? Cardiol Young. 2006 Jun;16(3):289-99. doi: 10.1017/S1047951106000400.\ (no transferability)

Elizabeth R Hammond, Audrey Kit Mei Foong, Norazlin Rosli, Dean E Morbeck. Should we freeze it? Agreement on fate of borderline blastocysts is poor and does not improve with a modified blastocyst grading system. Hum Reprod . 2020 May 1;35(5):1045-1053. doi: 10.1093/humrep/deaa060. (no transferability)

Ilker Seckiner, Serap Ulusam Seckiner, Sakip Erturhan, Ahmet Erbagci, Mehmet Solakhan, Faruk Yagci. The use of artificial neural networks in decision support in vesicoureteral reflux treatment. Urol Int . 2008;80(3):283-6. doi: 10.1159/000127342. (no transferability)

Tamang S, D Kopec, G Shagas, K Levy. Improving end of life care: an information systems approach to reducing medical errors. Stud Health Technol Inform. 2005; 114: 93-104 (preliminary results of prototype)

D Razzouk, J J Mari, I Shirakawa, J Wainer, D Sigulem. Decision support system for the diagnosis of schizophrenia disorders. Braz J Med Biol Res. 2006 Jan;39(1):119-28. doi: 10.1590/s0100-879x2006000100014 (low generalizeability)

Elpis V Pavlidou, Joanne M Williams. Implicit learning and reading: insights from typical children and children with developmental dyslexia using the artificial grammar learning (AGL) paradigm. Res Dev Disabil . 2014 Jul;35(7):1457-72. doi: 10.1016/j.ridd.2014.03.040. (educational only)

Quan H, Srinivasan D, Khosravi A. Incorporating Wind Power Forecast Uncertainties Into Stochastic Unit Commitment Using Neural Network-Based Prediction Intervals. IEEE Trans Neural Netw Learn Syst. 2015 Sep;26(9):2123-35. doi: 10.1109/TNNLS.2014.2376696. (no medicine)

Igor Kaiserman , Mordechai Rosner, Jacob Pe'er. Forecasting the prognosis of choroidal melanoma with an artificial neural network. Ophthalmology. 2005 Sep;112(9):1608. doi: 10.1016/j.ophtha.2005.04.008. (no transferability)

Matthias Ganzinger, Jens Schrodt, Petra Knaup. A Concept for Graph-Based Temporal Similarity of Patient Data. Stud Health Technol Inform. 2019; 264:138-142. doi: 10.3233/SHTI190199. (preliminary results)

Jure Demšar, Iztok Lebar Bajec. Evolution of Collective Behaviour in an Artificial World Using Linguistic Fuzzy Rule-Based Systems. PLoS One . 2017 Jan 3;12(1):e0168876. doi: 10.1371/journal.pone.0168876. eCollection 2017. (biological studies)

Hu Y, Wen JY, Li XL, Wang DZ, Li Y. A dynamic multimedia fuzzy-stochastic integrated environmental risk assessment approach for contaminated sites management. J Hazard Mater. 2013 Oct 15;261:522-33. doi: 10.1016/j.jhazmat.2013.08.009. (environmental)

John RA, Tiwari N, Patdillah MIB, Kulkarni MR, Tiwari N, Basu J, Bose SK, Ankit, Yu CJ, Nirmal A, Vishwanath SK, Bartolozzi C, Basu A, Mathews N. Self healable neuromorphic memtransistor elements for decentralized sensory signal processing in robotics. Nat Commun. 2020 Aug 12;11(1):4030. doi: 10.1038/s41467-020-17870-6. (only technical)

Hao Quan, Dipti Srinivasan, Abbas Khosravi. Incorporating Wind Power Forecast Uncertainties Into Stochastic Unit Commitment Using Neural Network-Based Prediction Intervals. IEEE Trans Neural Netw Learn Syst. 2015 Sep;26(9):2123-35. doi: 10.1109/TNNLS.2014.2376696. (environmental)

Jan Sosulski, Michael Tangermann. Extremely Reduced Data Sets Indicate Optimal Stimulation Parameters for Classification in Brain-Computer Interfaces. Conf Proc IEEE Eng Med Biol Soc. 2019 Jul;2019:2256-2260. doi: 10.1109/EMBC.2019.8857460. (technical)

Afshar P, Brown M, Maciejowski J, Wang H. Data-based robust multiobjective optimization of interconnected processes: energy efficiency case study in papermaking. IEEE Trans Neural Netw. 2011 Dec;22(12):2324-38. doi: 10.1109/TNN.2011.2174444. (technical)

Lichtenberg N, Eulzer P, Romano G, Brčić A, Karck M, Lawonn K, De Simone R, Engelhardt S. Mitral valve flattening and parameter mapping for patient-specific valve diagnosis. Int J Comput Assist Radiol Surg. 2020 Apr;15(4):617-627. doi: 10.1007/s11548-019-02114-w. (images improvement)

Annemie Heselmans, Bert Aertgeerts, Peter Donceel, Stijn Van de Velde, Peter Vanbrabant, Dirk Ramaekers. Human computation as a new method for evidence-based knowledge transfer in Web-based guideline development groups: proof of concept randomized controlled trial. Randomized Controlled Trial J Med Internet Res. 2013 Jan 17;15(1):e8. doi: 10.2196/jmir.2055. (methodology for guidelines development)

M Horino 1, M Hosoba, H Wani, N Oriuchi, M Tateno, T Inoue, Y Sasaki, H Igarashi, T Iizuka. Development and clinical application of an expert system for supporting diagnosis of 201Tl stress myocardial SPECT. Kaku Igaku. 1990 Feb;27(2):93-106. [Article in Japanese]

Coto Hernández I, Yang W, Mohan S, Jowett N. Label-free histomorphometry of peripheral nerve by stimulated Raman spectroscopy. Muscle Nerve. 2020 Jul;62(1):137-142. doi: 10.1002/mus.26895.

(technical)

Ma H. Mapping clause of Arden Syntax with HL7 and ASTM E 1238-88 standard. Int J Biomed Comput. 1995 Jan;38(1):9-21. doi: 10.1016/0020-7101(94)01029-z. (technical)

Coto Hernández I, Yang W, Mohan S, Jowett N. Label-free histomorphometry of peripheral nerve by stimulated Raman spectroscopy. Muscle Nerve. 2020 Jul;62(1):137-142. doi: 10.1002/mus.26895. (technical)

C P McDonald, R Cook, A Engel, S Robbins, I Rayfield, J A Barbara. Robotic selective sampling and total automation for anti-CMV screening. Transfus Med. 1999 Dec;9(4):301-5. doi: 10.1046/j.1365-3148.1999.00213.x. (no transferability)

Yi Z, Tan KK, Lee TH. Multistability analysis for recurrent neural networks with unsaturating piecewise linear transfer functions. Neural Comput. 2003 Mar;15(3):639-62. doi: 10.1162/089976603321192112. (technical)

Interpretive reporting to improve the effectiveness of clinical laboratory test results. An ECRI technology assessment. J Health Care Technol. 1986;2(4):269-82. (lab results interpretation)

Akbari Moornani K, Haeri M. On robust stability of linear time invariant fractional-order systems with real parametric uncertainties. ISA Trans. 2009 Oct;48(4):484-90. doi: 10.1016/j.isatra.2009.04.006. (mathematical)

Yu-Jun Zheng, Xiao-Han Zhou, Wei-Guo Sheng, Yu Xue, Sheng-Yong Chen. Generative adversarial network-based telecom fraud detection at the receiving bank. Neural Netw. 2018 Jun;102:78-86. doi: 10.1016/j.neunet.2018.02.015. (banking services)

Rodriguez-Ascaso A, Letón E, Muñoz-Carenas J, Finat C. Accessible mathematics videos for non-disabled students in primary education. PLoS One. 2018 Nov 28;13(11):e0208117. doi: 10.1371/journal.pone.0208117. (students education)

Helali F. How could you use the ergonomics 'knowhow' transfer management to enhance human working for sustainable improvements in industrially developing countries? Work. 2012;41 Suppl 1:2730-5. doi: 10.3233/WOR-2012-0517-2730. (labor forces)

Li S, Li A, Molina Lara DA, Gómez Marín JE, Juhas M, Zhang Y. Transfer Learning for Toxoplasma gondii Recognition. mSystems. 2020 Jan 28;5(1):e00445-19. doi: 10.1128/mSystems.00445-19. (no transferability)

Global Burden of Disease Cancer Collaboration, Fitzmaurice C, Abate D, Abbasi N et al. Global, Regional, and National Cancer Incidence, Mortality, Years of Life Lost, Years Lived With Disability, and Disability-Adjusted Life-Years for 29 Cancer Groups, 1990 to 2017: A Systematic Analysis for the Global Burden of Disease Study. JAMA Oncol. 2019 Dec 1;5(12):1749-1768. doi: 10.1001/jamaoncol.2019.2996. (burden of disease study)

Khalid S, Shahid M; Natasha, Bibi I, Sarwar T, Shah AH, Niazi NK. A Review of Environmental Contamination and Health Risk Assessment of Wastewater Use for Crop Irrigation with a Focus on Low and High-Income Countries. Int J Environ Res Public Health. 2018 May 1;15(5):895. doi: 10.3390/ijerph15050895 (environmental)

Zeleke AA, Worku AG, Demissie A, Otto-Sobotka F, Wilken M, Lipprandt M, Tilahun B, Röhrig R. Evaluation of Electronic and Paper-Pen Data Capturing Tools for Data Quality in a Public Health Survey in a Health and Demographic Surveillance Site, Ethiopia: Randomized Controlled Crossover Health Care Information Technology Evaluation. JMIR Mhealth Uhealth. 2019 Feb 11;7(2):e10995. doi: 10.2196/10995.

Alberto M R Dávila, Mário Steindel, Edmundo C Grisard. Tropical diseases, pathogens, and vectors biodiversity in developing countries: need for development of genomics and bioinformatics approaches. Ann N Y Acad Sci. 2004 Oct;1026:41-6. doi: 10.1196/annals.1307.005. (biological)

T N Anand, Linju M Joseph, A V Geetha, Joyita Chowdhury, Dorairaj Prabhakaran, Panniyammakal Jeemon. Task-sharing interventions for cardiovascular risk reduction and lipid outcomes in low- and middle-income countries: A systematic review and meta-analysis. J Clin Lipidol. May-Jun 2018;12(3):626-642. doi: 10.1016/j.jacl.2018.02.008. (no recommendations)

Karla Hernandez-Villafuerte, Ryan Li, Karen J Hofman. Bibliometric trends of health economic evaluation in Sub-Saharan Africa. Global Health. 2016 Aug 24;12(1):50. doi: 10.1186/s12992-016-0188-2. (bibliometric analysis)

Dellepiane N, Pagliusi S; Registration Experts Working Group. Challenges for the registration of vaccines in emerging countries: Differences in dossier requirements, application and evaluation processes. Vaccine. 2018 Jun 7;36(24):3389-3396. doi: 10.1016/j.vaccine.2018.03.049. (clinical trials discussion)

Shahnavaz H. Role of ergonomics in the transfer of technology to industrially developing countries. Ergonomics. 2000 Jul;43(7):903-7. doi: 10.1080/001401300409099. (ergonimics)

Mathias Flume, Marc Bardou, Stefano Capri, Oriol Sola-Morales, David Cunningham, Lars-Ake Levin, Maarten J Postma, Nicolas Touchot. Approaches to manage 'affordability' of high budget impact medicines in key EU countries .J Mark Access Health Policy. 2018 Jun 8;6(1):1478539. doi: 10.1080/20016689.2018.1478539. eCollection 2018. (no developing coutries)

Vivien Davis Tsu, Michael J Free. Using technology to reduce maternal mortality in low-resource settings: challenges and opportunities. J Am Med Womens Assoc (1972). Summer 2002;57(3):149-53. (no AI)

P Heimann. Technology for health. Afr Health. 1994 Mar;(Spec No):14-7. (no transferability)

Sacre H, Tawil S, Hallit S, Sili G, Salameh P. Mandatory continuing education for pharmacists in a developing country: assessment of a three-year cycle. Pharm Pract (Granada). 2019 Jul-Sep;17(3):1545. doi: 10.18549/PharmPract.2019.3.1545. (no transferability)

Mishori R, Anastario M, Naimer K, Varanasi S, Ferdowsian H, Abel D, Chugh K. mJustice: Preliminary Development of a Mobile App for Medical-Forensic Documentation of Sexual Violence in Low-Resource Environments and Conflict Zones. Glob Health Sci Pract. 2017 Mar 28;5(1):138-151. doi: 10.9745/GHSP-D-16-00233. (forensic documentation)

Ullah R, Asghar R, Baqar M, Mahmood A, Ali SN, Sohail M, Schäfer RB, Eqani SAMAS. Assessment of organochlorine pesticides in the Himalayan riverine ecosystems from Pakistan using passive sampling techniques. Environ Sci Pollut Res Int. 2019 Feb;26(6):6023-6037. doi: 10.1007/s11356-018-3987-6 (ecological)

Benjamin O Anderson 1, Cheng-Har Yip, Robert A Smith, Roman Shyyan, Stephen F Sener, Alexandru Eniu, Robert W Carlson, Edward Azavedo, Joe Harford. Guideline implementation for breast healthcare in low-income and middle-income countries: overview of the Breast Health Global Initiative Global Summit 2007. Cancer. 2008 Oct 15;113(8 Suppl):2221-43. doi: 10.1002/cncr.23844. (no transferability)

Huck-Soo L, Richardson S. Ergonomics in industrially developing countries: a literature review. J Hum Ergol (Tokyo). 2012 Dec;41(1-2):1-16. (ergonomic)

Holm R, Wandschneider P, Felsot A, Msilimba G. Achieving the sustainable development goals: a case study of the complexity of water quality health risks in Malawi. J Health Popul Nutr. 2016 Jul 15;35(1):20. doi: 10.1186/s41043-016-0057-x. (ecological)

Raza MW, Kazi BM, Mustafa M, Gould FK. Developing countries have their own characteristic problems with infection control. J Hosp Infect. 2004 Aug;57(4):294-9. doi: 10.1016/j.jhin.2004.03.019. (no transferability)

M Hirschfeld. The "Canadian model" in the international context. Healthc Pap. Fall 2000;1(4):103-7, discussion 109-12. doi: 10.12927/hcpap..17360. (only comment)

P D Lee. The role of appropriate medical technology procurement and user maintenance instructions in developing countries. J Clin Eng. Sep-Oct 1995;20(5):407-13. doi: 10.1097/00004669-199509000-00016. (procurement)

M López-Cervantes, L L Tirado-Gómez, E de Icaza-del Río, L Durán-Arenas. [Medical technology and health. A lot of crunching but few nuts?][Article in Spanish] Rev Invest Clin. Mar-Apr 2000;52(2):203-10.

Homma A, Knouss RF. The transfer of vaccine technology to developing countries. The Latin American experience. Int J Technol Assess Health Care. 1994 Winter;10(1):47-54. doi: 10.1017/s0266462300013970. (manufacturing)

Xiaochu Yu, Jingmei Jiang, Changwei Liu, Keng Shen, Zixing Wang, Wei Han, Xingrong Liu, Guole Lin, Ye Zhang, Ying Zhang, Yufen Ma, Haixin Bo, Yupei Zhao Protocol for a multicentre, multistage, prospective study in China using system-based approaches for consistent improvement in surgical safety. BMJ Open. 2017 Jun 15;7(6):e015147. doi: 10.1136/bmjopen-2016-015147. (no transferability)

Gong L, Yu P, Zheng H, Gu W, He W, Tang Y, Wang Y, Dong Y, Peng X, She Q, Xie L, Chen L. Comparative genomics for non-O1/O139 Vibrio cholerae isolates recovered from the Yangtze River Estuary versus V. cholerae representative isolates from serogroup O1. Mol Genet Genomics. 2019 Apr;294(2):417-430. doi: 10.1007/s00438-018-1514-6. Epub 2018 Nov 28. (virology)

Vossberg A. The choice of prosthetic and orthotic technique for less developed countries: analysis and perspectives in Colombia. Prosthet Orthot Int. 1988 Aug;12(2):96-100. doi: 10.3109/03093648809078206. (no transferability)

Lema G, Mesfun MG, Eshete A, Abdeta G. Assessment of status of solid waste management in Asella town, Ethiopia. BMC Public Health. 2019 Sep 12;19(1):1261. doi: 10.1186/s12889-019-7551-1. (ecological)

Forjuoh SN. Traffic-related injury prevention interventions for low-income countries. Inj Control Saf Promot. 2003 Mar-Jun;10(1-2):109-18. doi: 10.1076/icsp.10.1.109.14115. (no transferability)

Japiong KB, Asiamah G, Owusu-Dabo E, Donkor P, Stewart B, Ebel BE, Mock CN. Availability of resources for emergency care at a second-level hospital in Ghana: A mixed methods assessment. Afr J Emerg Med. 2016 Mar;6(1):30-37. doi: 10.1016/j.afjem.2015.06.006. Epub 2015 Sep 4. (only national, no transferability)

Ikeda AJ, Grabowski AM, Lindsley A, Sadeghi-Demneh E, Reisinger KD. A scoping literature review of the provision of orthoses and prostheses in resource-limited environments 2000-2010. Part one: considerations for success. Prosthet Orthot Int. 2014 Aug;38(4):269-86. doi: 10.1177/0309364613500690. (dental medicine, no transferability)

J Ovretveit. Would it work for us? Learning from quality improvement in Europe and beyond. Jt Comm J Qual Improv . 1997 Jan;23(1):7-22. doi: 10.1016/s1070-3241(16)30290-5. (quality assurance)

Taylhardat AR, Zilinskas RA. Agenda 21: biotechnology at the United Nations Conference on Environment and Development. Biotechnology (N Y). 1992 Apr;10(4):402-4. doi: 10.1038/nbt0492-402. (no tranferability)

Coleman R, Gill G, Wilkinson D. Noncommunicable disease management in resource-poor settings: a primary care model from rural South Africa. Bull World Health Organ. 1998;76(6):633-40. (no tranferability)

Stewart BT, Gyedu A, Quansah R, Addo WL, Afoko A, Agbenorku P, Amponsah-Manu F, Ankomah J, Appiah-Denkyira E, Baffoe P, Debrah S, Donkor P, Dorvlo T, Japiong K, Kushner AL, Morna M, Ofosu A, Oppong-Nketia V, Tabiri S, Mock C. District-level hospital trauma care audit filters: Delphi technique for defining context-appropriate indicators for quality improvement initiative evaluation in developing countries. Injury. 2016 Jan;47(1):211-9. doi: 10.1016/j.injury.2015.09.007.28. (QA)

Kayyal M, Gibbs T. Applying a quality assurance system model to curriculum transformation: transferable lessons learned. Med Teach. 2012;34(10):e690-7. doi: 10.3109/0142159X.2012.687486. (QA-education)

Serpa-Flórez F.Technology transfer to developing countries. Lessons from Colombia. Int J Technol Assess Health Care. 1993 Spring;9(2):233-7. doi: 10.1017/s0266462300004451. (no transferability just planning)

Benagiano G, Diczfalusy E. Research on human reproduction and the United Nations. S Afr Med J. 1995 May;85(5):370-3. (no transferability)

De Cock KM, Ekpini E, Gnaore E, Kadio A, Gayle HD. The public health implications of AIDS research in Africa. JAMA. 1994 Aug 10;272(6):481-6. doi: 10.1001/jama.272.6.481. (no transferability)

Orem J, Otieno MW, Banura C, Katongole-Mbidde E, Johnson JL, Ayers L, Ghannoum M, Fu P, Feigal EG, Black J, Whalen C, Lederman M, Remick SC. Capacity building for the clinical investigation of AIDS malignancy in East Africa. Cancer Detect Prev. 2005;29(2):133-45. doi: 10.1016/j.cdp.2004.10.001 (clinical research)

Friedman EA. Facing the reality: the world cannot afford uremia therapy at the start of the 21st century. Artif Organs. 1995 May;19(5):481-5. doi: 10.1111/j.1525-1594.1995.tb02363.x. (no transferability)

W Vandersmissen. Availability of quality vaccines: the industrial point of view. Vaccine. 1992;10(13):955-7. doi: 10.1016/0264-410x(92)90333-f. (supply policy)

Burney MI. Transferring manufacturing technology. Producing essential vaccines in Pakistan. Int J Technol Assess Health Care. 1993 Summer;9(3):397-406. doi: 10.1017/s0266462300004657. (manufacturing)

Mahmud SG, Shamsuddin SA, Ahmed MF, Davison A, Deere D, Howard G. Development and implementation of water safety plans for small water supplies in Bangladesh: benefits and lessons learned. J Water Health. 2007 Dec;5(4):585-97. doi: 10.2166/wh.2007.045. (environmental)

A Abbott 1, P D Abel, D W Arnold, A Milne. Cost-benefit analysis of the use of TBT: the case for a treatment approach. Sci Total Environ. 2000 Aug 21;258(1-2):5-19. doi: 10.1016/s0048-9697(00)00505-2. (outside of medical area)

B F Stanton 1, A M Fitzgerald, X Li, H Shipena, I B Ricardo, J S Galbraith, N Terreri, J Strijdom, V Hangula-Ndlovu, J Kahihuata. HIV risk behaviors, intentions, and perceptions among Namibian youth as assessed by a theory-based questionnaire. AIDS Educ Prev. 1999 Apr;11(2):132-49. (no transferability)

Halstead SB. Tissue culture-based rabies vaccines: vaccine production technology transfer. Rev Infect Dis. 1988 Nov-Dec;10 Suppl 4:S764-5. doi: 10.1093/clinids/10.supplement_4.s764. (biological)

Ho NK. Relevance of neonatal care in developing countries. Singapore Med J. 1999 Sep;40(9):558-60. (No abstract available.)

Smith-Jackson TL, Essuman-Johnson A. Cultural ergonomics in Ghana, West Africa: a descriptive survey of industry and trade workers' interpretations of safety symbols. Int J Occup Saf Ergon. 2002;8(1):37-50. doi: 10.1080/10803548.2002.11076513. (ecology)

András Inotai, Tamás Ágh, Alexei Willem Karpenko, Antal Zemplényi, Zoltán Kaló. Behind the subcutaneous trastuzumab hype: evaluation of benefits and their transferability to Central Eastern European countries. Expert Rev Pharmacoecon Outcomes Res. 2019 Apr;19(2):105-113. doi: 10.1080/14737167.2019.1554437. (medicinal products dosage form comparison)

Marcell Csanádi, András Inotai, Oleksandra Oleshchuk, Oksana Lebega, Brodovskaya Alexandra, Oresta Piniazhko, Bertalan Németh, Zoltán Kaló. Health Technology Assessment Implementation in Ukraine: Current Status and Future Perspectives Int J Technol Assess Health Care. 2019;35(5):393-400. doi: 10.1017/S0266462319000679. (educational programs on HTA at national level)

Bertalan Németh, Zoltán Kaló. European cooperation in health technology assessment implementation: the perspective of Central and Eastern European countries. J Comp Eff Res. 2020 Jun;9(9):599-602. doi: 10.2217/cer-2020-0062. (cooperation discussion – editorial comment)

Zoltán Vokó, Renáta Németh, László Nagyjánosi, György Jermendy, Gábor Winkler, Tibor Hídvégi, Zoltán Kalotai, Zoltán Kaló. Mapping the Nottingham Health Profile onto the Preference-Based EuroQol-5D Instrument for Patients with Diabetes. Value Health Reg Issues. 2014; 4:31-36. doi: 10.1016/j.vhri.2014.06.002. (national study)

Fasseeh A, Karam R, Jameleddine M, George M, Kristensen FB, Al-Rabayah AA, Alsaggabi AH, El Rabbat M, Alowayesh MS, Chamova J, Ismail A, Abaza S, Kaló Z. Implementation of Health Technology Assessment in the Middle East and North Africa: Comparison Between the Current and Preferred Status. Front Pharmacol. 2020;11:15. doi: 10.3389/fphar.2020.00015. eCollection 2020. (middle east and north Africa countries)

Kaló Z, Landa K, Doležal T, Vokó Z. Transferability of National Institute for Health and Clinical Excellence recommendations for pharmaceutical therapies in oncology to Central-Eastern European countries. Eur J Cancer Care (Engl). 2012;21(4):442-9. doi: 10.1111/j.1365-2354.2012.01351.x. (pharmaceuticals and health care reports transferability)

Mandrik O, Knies S, Kaló Z, Severens JL. Transferability of Economic Evaluations To Central and Eastern European and Former Soviet Countries. Value Health. 2014;17(7):A443-4. doi: 10.1016/j.jval.2014.08.1172. (oriented to national content)

Bertalan Németh, Wim Goettsch, Finn Børlum Kristensen, Oresta Piniazhko, Mirjana Huić, Tomáš Tesař, Dragana Atanasijevic, Iga Lipska, Zoltán Kaló. The transferability of health technology assessment: the European perspective with focus on central and Eastern European countries. Expert Rev Pharmacoecon Outcomes Res. 2020;20(4):321-330. doi: 10.1080/14737167.2020.1779061.

Kamran Ahmed, Reenam Khan, Alexandre Mottrie, Catherine Lovegrove, Ronny Abaza, Rajesh Ahlawat, Thomas Ahlering, Goran Ahlgren, Walter Artibani, Eric Barret, Xavier Cathelineau, Ben Challacombe, Patrick Coloby, Muhammad S Khan, Jacques Hubert, Maurice Stephan Michel, Francesco Montorsi, Declan Murphy, Joan Palou, Vipul Patel, Pierre-Thierry Piechaud, Hendrik Van Poppel, Pascal Rischmann, Rafael Sanchez-Salas, Stefan Siemer, Michael Stoeckle, Jens-Uwe Stolzenburg, Jean-Etienne Terrier, Joachim W Thüroff, Christophe Vaessen, Henk G Van Der Poel, Ben Van Cleynenbreugel, Alessandro Volpe, Christian Wagner, Peter Wiklund, Timothy Wilson, Manfred Wirth, Jörn Witt, Prokar Dasgupta. Development of a standardised training curriculum for robotic surgery: a consensus statement from an international multidisciplinary group of experts. BJU Int. 2015 Jul;116(1):93-101. doi: 10.1111/bju.12974 (therapeutic guideline creation)

Ortmann O, J Torode, W Schmiegel, C Thomssen, M Hakama, P Basu, U Ringborg, U Helbig, participants of the ERTM. Knowledge transfer as a tool towards improvement of cancer care in low- and middle-income countries. 6th European Roundtable Meeting (ERTM), June 14th, 2019, Berlin, Germany. J Cancer Res Clin Oncol. 2020 Jul;146(7):1813-1818. doi: 10.1007/s00432-020-03209-7. (opinions and experience exchange)

Coe GA, D Banta. Health care technology transfer in Latin America and the Caribbean. Int J Technol Assess Health Care. Spring 1992;8(2):255-67. doi: 10.1017/s0266462300013489 (not CEE)

Teerawattananon Y, Tantivess S, Yamabhai I, Tritasavit N, Walker DG, Cohen JT, Neumann PJ. The influence of cost-per-DALY information in health prioritisation and desirable features for a registry: a survey of health policy experts in Vietnam, India and Bangladesh. Health Res Policy Syst. 2016; 14 (1): 86. doi: 10.1186/s12961-016-0156-6. (not CEE)

Bonair A, P Rosenfield, K Tengvald. Medical technologies in developing countries: issues of technology development, transfer, diffusion and use. Soc Sci Med. 1989;28(8):769-81. doi: 10.1016/0277-9536(89)90106-8. (policy paper)

Arnold J, Alex Davis, Baruch Fischhoff, Emmanuelle Yecies, Jon Grace, Andrew Klobuka, Deepika Mohan, Janel Hanmer. Comparing the predictive ability of a commercial artificial intelligence early warning system with physician judgement for clinical deterioration in hospitalised general internal medicine patients: a prospective observational study. BMJ Open. 2019 Oct 10;9(10):e032187. doi: 10.1136/bmjopen-2019-032187. (do not discuss transferability)

Tuijn CJ, Hoefman BJ, van Beijma H, Oskam L, Chevrollier N. Data and image transfer using mobile phones to strengthen microscopy-based diagnostic services in low and middle income country laboratories. PLoS One. 2011;6(12):e28348. doi: 10.1371/journal.pone.0028348. (only technical issues)

Chizzali-Bonfadin C, K P Adlassnig, W Koller. MONI: an intelligent database and monitoring system for surveillance of nosocomial infections. Medinfo. 1995;8 Pt 2:1684. (do not discuss transferability)
